# Uncertainty and certainty in cellular dynamics

**DOI:** 10.3389/fgene.2013.00068

**Published:** 2013-04-24

**Authors:** Kumar Selvarajoo

**Affiliations:** ^1^Institute for Advanced Biosciences, Keio UniversityTsuruoka, Japan; ^2^Graduate School of Media and Governance, Keio UniversityFujisawa, Japan

Understanding the origins and diversity of life has been arguably the most sought after desire since the birth of mankind. For higher organisms, a single cell origin diversifying into multiple cell types along the differentiation trajectories is very intriguing. Although numerous works on stem cells and cell differentiation research have elucidated indispensible details of crucial cellular markers necessary for diversification, whether randomness or determinism governs cell fate decision is still debatable (Losick and Desplan, 2008; Jullien et al., 2011).

In Charles Darwin's evolutionary theory, natural selection refers to a gradual adaptation of species to its environment over long periods of time through a non-random process (Darwin, 1859; Beddall, 1968; Wright, 1968). That is, Darwin believed that some sort of memory states exist in living system that is capable to invoke survivability under drastic environmental changes over time. This idea is in contrast to his predecessors, who believed in “chance” for species diversification for survival. So, at cellular level, is probability or non-random process crucial for a cell's survival to environmental changes?

**Figure 1:**
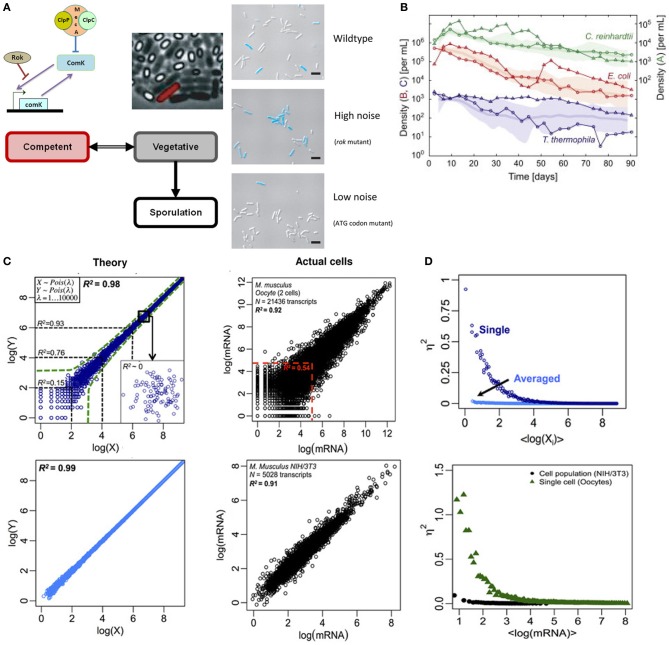
**Noisy single cell and deterministic population level responses. (A)** Left: switching between vegetative (gray) and competent (red) state in *B. subtilis* is guided by the level of stochastic noise in the transcription of comK mRNA. Right: live image where competent cell (blue) proportion increases with stochastic noise. Note that *rok* mutant exhibits an increased transcriptional bursting in comK mRNA compared to WT strain, and the mutant in ATG initiation codon reduces its translational efficiency. Figure modified from Süel et al. (2006) and Maamar et al. (2007). **(B)** Population dynamics of *C. reinhardtii*, *E. coli*, and *T. thermophila* kept under constant light and temperature display random walk over bounded average response. Figure adapted from Hekstra and Leibler (2012). **(C)** Whole transcriptome correlations. Left panels indicate theoretically generated data, and right panels show actual cells' data (top: oocytes, bottom: NIH/3T3 cell culture). The correlation of 30 random sets of theoretical expressions data averaged (bottom left) eliminates stochastic noise (due to canceling of positive and negative deviations), and almost shows the population level correlation of NIH/3T3 cell cultures (bottom right). Figure modified from Piras et al. (2012). **(D)** Noise (η^2^) vs. expressions (in natural logarithm). Theory (top) and actual (bottom) data show stochastic noise reduces with expression levels for single cells. Cell populations show near zero stochastic noise. Figure modified from Piras et al. (2012).

Recent works in *Bacillus subtilis* have shown that biological noise or randomness in transcriptional machinery is crucial for controlling cell fate decision. Depending on the amount of nutrient available, *B. subtilis* survives in three modes: vegetative, competent, and sporulative. A key molecule for switching states between vegetative and competence is the transcription factor ComK. In nutrient deficient conditions, *B. subtilis* survives in competent state by DNA uptake from the surrounding, facilitated by ComK which support the construction of DNA-binding and uptake apparatus (van Sinderen et al., 1995). In other words, lower concentration of ComK refers to vegetative state while higher concentration leads to competence, with an unknown threshold level switching between the states. However, a population of *B. subtilis* in nutrient deficient conditions does not deterministically show all cells becoming competent. Rather, a mixture of vegetative, competent, and sporulated cells exists (Figure 1A, left).

To understand the cell fate control mechanisms of *B. subtilis*, Dubnau and colleagues regulated the expressions of comK mRNA by constructing synthetic strains with low and high stochastic noise. They showed that *rok* mutants, with high-levels of stochastic bursting, shift the threshold of ComK concentration lower thereby favoring competence (Figure 1A, right) (Maamar et al., 2007). That is, although *B. subtilis* can exist in bistable states under nutrient-limited conditions, the phenotypic heterogeneity observed in the population can be controlled by the level of randomness in single cell dynamics. Other works controlling different components of the comK transcriptional machinery have revealed the importance of noise at different scales in the heterogeneous behavior of *B. subtilis* (Süel et al., 2006; Locke et al., 2011).

The intestinal cell fate process from early embryonic lineage in wildtype *Caenorhabditis elegans* has been considered deterministic and invariant. The transcription factor SKN-1 plays an important role in the developmental networks of intestinal specification, where the cell fate decision is dependent on the expression levels of *elt-2*. *Elt-2* is activated by *end-1* through *skn-1*, *med-1/2*, and *end-3* in the transcriptional networks (see Figure 2B of reference Raj et al., 2010). Oudenaarden and colleagues examined the incomplete penetrance of *C. elegans* using *skn-1* mutant strains where some embryos failed in the development of intestinal cells, while others became intestinal precursors in probabilistic manner (Raj et al., 2010). The differentiation phenotype of *skn-1* mutant *C. elegans* was determined when the fluctuating expression of *end-1* reached a certain threshold to switch *elt-2* “ON” for differentiation. Especially, the mutation of *skn-1* caused the *med-1/2* and *end-3* transcripts to essentially diminish and effectively be removed from the intestinal gut gene network. As a result, the modified network increased the variability in *end-1* significantly compared to wildtype which was crucial for switching the intestinal cell differentiation outcome. In other words, stochastic variability in gene expressions can be buffered by certain molecules such as *med-1/2* and *end-3* (acting as noise suppressors) whose removal can lead to pronounced phenotypic variation.

The works on *B. subtilis* and *C. elegans* suggest that randomness in genetic circuits creates uncertainty in whether an individual cell or organism, within a clonal population, will change its cell fate to a given environmental perturbation. If living cells are guided purely by random events, crucial for generating phenotypic heterogeneity, how do precursor cells stably differentiate along predetermined trajectory? Alternatively, how does the immune system robustly neutralizes invading pathogens in higher organisms? In line with the issue, Nobel laureate Gurdon and colleagues recently question the role of randomness in the early embryonic development process where the nuclear reprogramming by eggs and oocytes occurs in an ordered and precise timing (Jullien et al., 2011). However, they do acknowledge that the effects of epigenetics and stochasticity could affect the efficiency of the reprogramming process. Biology, thus, possess the capability to produce deterministic outcome based on past events.

To understand how historical events could shape future outcome, Hekstra and Leibler carefully constructed a microbial closed system with well-controlled initial conditions of *C. reinhardtii*, *E. coli* and *T. thermophila* and studied their growth dynamics over 90 days (Hekstra and Leibler, 2012). Notably, despite variability at individual level, the population dynamics revealed emergent average deterministic response following simple statistical laws for all three species (Figure 1B). Furthermore, the fluctuations around the average dynamics displayed power-law, consistent with geometric random walk. In other words, although individual cells display fluctuating response, their global means follow deterministic paths.

To probe deeper into the issue of randomness (noise) and determinism, we investigated the whole transcriptome of two biological species: single oocytes and NIH/3T3 cell culture (Piras et al., 2012). For single cells (oocytes), Pearson correlation analysis between samples showed high overall correlation (*R*^2^ = 0.98). However, for species with low copy numbers, the correlations were significantly lower [e.g., *R*^2^ < 0.54 for log(X) <5] (Figure 1C, upper right). From theory, the scatter for lower expressions is due to stochastic noise (Figure 1C, upper left) (Piras et al., 2012). On the other hand, for cell population (NIH/3T3), the Pearson correlation is high but, distinctively, the data show minimal scatter for lower expressions indicating lack of randomness (Figure 1C, bottom right).

Correspondingly, when noise was quantified, the stochastic noise for single cells is dominant for lower gene expressions and it approaches zero as expression levels increase (Figure 1D). However, the stochastic noise for cell population is almost absent across all expression levels. This result is likely due to the canceling of positive and negative noises across the entire transcriptome, especially for cell populations. That is, cell populations show deterministic average response, or attractor state, where single molecule noises are eliminated, even for lowly expressed genes, when investigated through global transcriptome. This may be compared with the law of large numbers, where the uncertainty of individual trial (gene) is averaged across multiple trials (transcriptome) to reveal the mean value (deterministic response).

In summary, biological responses should be interpreted carefully by studying their dynamical evolution and scale. As seen from single cell system or small-scale local cell differentiation networks, stochastic fluctuations are necessary to induce probabilistic differentiation between survival states. Moreover, the specific changes in cell states and its occurrences remain elusive indicating that cell fate decision is controlled by memory-less random process. On the other hand, well-coordinated response of cell populations, such as cells responsible for growth or immune response, demonstrates that cells are able to invoke global response based on past events. So, how does biology embed both random events and deterministic response?

We believe that the origin for randomness arises from single cells where the issue of stochasticity is dominant for the lowly expressed molecules. Notably, for cell population, the effect of stochasticity is almost absent when viewed globally such as transcriptome-wide. Overall, as observed in other physical sciences, biology is a complex system possessing both microscopic random and macroscopic deterministic dynamics. It is, thus, necessary to distinguish the two fundamental characteristics for interpreting the complex dynamic response of living systems.
